# Methodology for Simulating 5G and GNSS High-Accuracy Positioning

**DOI:** 10.3390/s18103220

**Published:** 2018-09-24

**Authors:** José A. del Peral-Rosado, Jani Saloranta, Giuseppe Destino, José A. López-Salcedo, Gonzalo Seco-Granados

**Affiliations:** 1 Department of Telecommunications and Systems Engineering, Universitat Autònoma de Barcelona, 08193 Cerdanyola del Vallès, Spain; jose.salcedo@uab.cat (J.A.L.-S.); gonzalo.seco@uab.cat (G.S.-G.); 2 Centre for Wireless Communications, University of Oulu, 90014 Oulu, Finland; jani.saloranta@oulu.fi (J.S.); giuseppe.destino@oulu.fi (G.D.); 3 Centre for Telecommunication Research, King’s College London, London WC2 R2LF, UK

**Keywords:** GNSS, 5G positioning, cmWave, mmWave, hybrid localization

## Abstract

This paper focuses on the exploitation of fifth generation (5G) centimetre-wave (cmWave) and millimetre-wave (mmWave) transmissions for high-accuracy positioning, in order to complement the availability of Global Navigation Satellite Systems (GNSS) in harsh environments, such as urban canyons. Our goal is to present a representative methodology to simulate and assess their hybrid positioning capabilities over outdoor urban, suburban and rural scenarios. A novel scenario definition is proposed to integrate the network density of 5G deployments with the visibility masks of GNSS satellites, which helps to generate correlated scenarios of both technologies. Then, a generic and representative modeling of the 5G and GNSS observables is presented for snapshot positioning, which is suitable for standard protocols. The simulations results indicate that GNSS drives the achievable accuracy of its hybridisation with 5G cmWave, because non-line-of-sight (NLoS) conditions can limit the cmWave localization accuracy to around 20 m. The 5G performance is significantly improved with the use of mmWave positioning with dominant line-of-sight (LoS) conditions, which can even achieve sub-meter localization with one or more base stations. Therefore, these results show that NLoS conditions need to be weighted in 5G localization, in order to complement and outperform GNSS positioning over urban environments.

## 1. Introduction

Precise and reliable localization is a topic of high interest for autonomous and unmanned vehicles [1], such as self-driving cars and drones. Indeed, the automotive industry demands positioning accuracies at the cm-level, in order to enable vehicular use cases based on automated driving and road safety [2]. Current localization technologies used for these critical applications are based on Global Navigation Satellite Systems (GNSS) for absolute positioning, and the combination of radars, cameras and inertial sensors for relative positioning. Nonetheless, the high implementation cost of these on-board sensors may prevent their adoption in certain applications. Thus, wireless networks dedicated for vehicular-to-everything (V2X) communications can also be exploited for positioning purposes. This is the case of fifth generation (5G) cellular networks, whose disruptive technologies are expected to enable high-accuracy localization.

The combination of GNSS and cellular networks has attracted special attention along the different network generations [3]. Cellular systems are typically considered to complement the lack of GNSS visibility in urban environments, as discussed in [4]. Most of these hybrid algorithms are based on the extended Kalman filter (EKF) or particle filter, as in [5,6]. These navigation solutions are necessary to filter noisy cellular observables over time. Furthermore, the cellular propagation channels are dominated by non-line-of-sight (NLoS) conditions and dense multipath. As studied in [7], the position errors are above 63 m on the 95% of the cases with 20-MHz Long Term Evolution (LTE) signals from field measurements. Therefore, the 5G new radio (NR) features [3,8], such as wideband signals, massive antenna arrays, millimetre wave (mmWave) transmissions, ultra-dense networks and device-to-device (D2D) communications, are expected to significantly enhance the hybrid positioning performance with GNSS. These NR features introduces high-accuracy ranging and angle measurements with a high network density, which are envisaged to achieve high-accuracy positioning. In addition, the 5G centimetre-wave (cmWave) transmissions with extended bandwidths are also of interest in macro-cell deployments. In this sense, the 3GPP standard has just approved a new study item on 5G NR positioning [9]. However, there is a limited literature on the integration of GNSS and 5G technologies, such as in [10].

The objective of this work is to present a reproducible methodology for the representative simulation of 5G and GNSS technologies in realistic scenarios. This methodology helps to characterize the effects of 5G cmWave and mmWave observables on GNSS positioning. However, to the best of the authors’ knowledge, current models are either too complex, such as those based on ray-tracing models as in [6], or too generic, such as in the LTE standard in [11]. Therefore, a novel methodology is proposed to couple the network density with the GNSS visibility masks. This methodology is then used to assess the achievable positioning performance of cmWave and mmWave technologies, with insights on the current limitations and promising features of 5G. The standardization of 5G positioning in [12] is expected to support dedicated network resources, i.e., positioning pilot signals, over short periods of time, in order to fulfil the stringent latency requirements (i.e., a position fix in less than 500 ms). This leads to the study of snapshot localization algorithms instead of navigation algorithms, where the former only uses one observable per GNSS satellite or 5G base station (BS) for positioning. This snapshot approach is especially suitable for standard protocols with periodical or on-demand positioning occassions, and to reduce the power consumption. The observables are only time-of-arrival (ToA) measurements for GNSS and cmWave positioning, while stand-alone mmWave positioning is based on ToA, angle-of-arrival (AoA) and angle-of-departure (AoD) estimates. The proposed methodology and the snapshot approach are applicable to outdoor use cases with high-accuracy positioning requirements at periodic or triggered events, such as the location-based services, emergency and mission critical applications or road-related uses cases defined in [1]. Thus, this contribution is especially convenient for the standardization of 5G NR positioning, in order to provide realistic and reproducible hybrid assessments.

The structure of the paper is as follows. The novel scenario definition is described in Section 2, with the relation between network density and GNSS visibility. The GNSS, cmWave and mmWave observables are characterized in Section 3. Then, the positioning algorithm is defined in Section 4. The simulation results are discussed in Section 5. Finally, the conclusions and future work are drawn in Section 6.

## 2. Scenario Definition

The evaluation of the localization improvements based on the combination of GNSS and cellular technologies requires a generic and representative framework, which does not specifically favors one technology. This approach is typically followed by standardization bodies that define reproducible scenarios in order to evaluate candidate specifications. For instance, scenario conditions for GNSS-based localization are defined in ETSI TS 103 246-3 [13] and 3GPP TS 36.171 [14], and for LTE-based localization in 3GPP TR 37.857 [11]. However, current standards do not define scenarios to evaluate the positioning performance of hybrid GNSS and cellular solutions. Thus, this section introduces a novel scenario definition to assess the stand-alone positioning capabilities of both technologies, and the enhancements of the complementary use of cellular networks to GNSS. The main scenarios are defined for outdoor urban, suburban and rural environments, while indoor scenarios are not considered in this work due to the lack of GNSS satellite visibility.

### 2.1. Network of Cellular Transmitters

Cellular systems are based on heterogeneous networks with different cell sizes, technologies and operation bands. This study focuses on the 5G positioning capabilities based on cmWave and mmWave deployments:

- **5G cmWave networks**: The 5G cmWave networks operate at frequency bands between 450 MHz and 6 GHz [15]. Due to the limited available spectrum, there is a reduced system bandwidth of up to 100 MHz, which can be improved with carrier aggregation. Thanks to the favorable propagation conditions at sub-6 GHz frequencies, rural macro cells achieve a large coverage area. According to [16], the inter-site distance (ISD) between rural macro BSs is defined to 1732 m or 5000 m. In urban areas, the ISD of the macro cells is reduced to 500 m, and hotspots are covered with small cells or micro sites of ISD equal to 200 m [16].
- **5G mmWave networks**: The 5G mmWave networks are defined for the frequency range (FR) from 24.25 GHz to 52.6 GHz, where large system bandwidths between 50 and 400 MHz can be allocated [15]. These networks are deployed over small cells, which may be co-located with micro cmWave sites or dedicated mmWave sites, in order to cope with very high communications demands over specific urban areas. The high attenuation losses at mmWave frequencies can be overcome thanks to massive antenna arrays [17] and innovative array signal processing [18].

Cellular networks are typically evaluated in the standard with generic scenarios. As described in [16], each network topology, i.e., urban micro, urban macro and rural macro, is simulated with a hexagonal cell layout of 19 three-sectorial sites and the corresponding ISD. These topologies can also be combined, such as in [11], where clusters of small cells are randomly and uniformly distributed per macro cell. However, in a realistic deployment, the density of BSs is mainly determined by the amount of potential users and topography of a certain area. For instance, suburban areas with a low building height have a low density of BSs, while a high density of BSs is considered in populated urban areas with a high-building profile. Furthermore, the location of BSs and their geometry have a significant relevance on the positioning analysis. Thus, realistic network deployments can be considered to improve the representativity of the simulation scenario. These deployments can be obtained from a public database of commercial cellular transmitters, e.g., from [19], and the confidentiality of the network can still be preserved by using only local coordinates.

**Figure 1:**
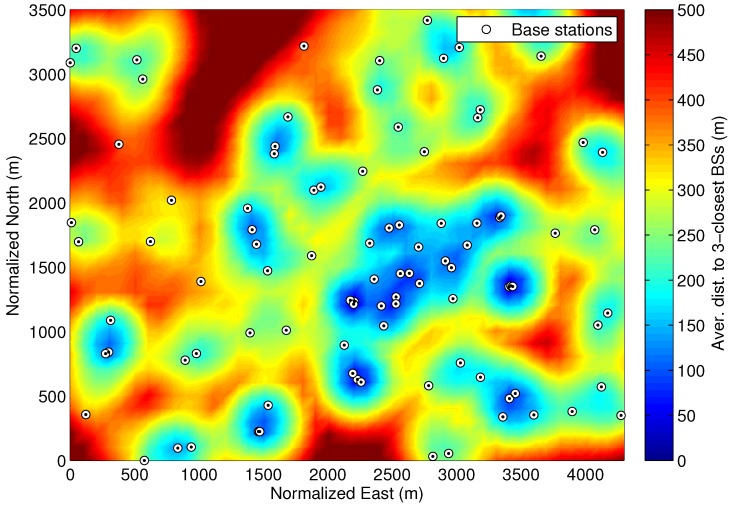
Average distance to the three closest BSs of a representative LTE network deployment.

A representative example of network deployment from [19] is shown in Figure 1, where the location of the cellular BSs is indicated with circles. In this example deployment, the minimum distance between two BS locations is equal to 10 m (i.e., for micro cells). The network density is here characterized by computing the average distance from a certain receiver position to the three closest BSs, i.e., $d_{\mathrm{av},3\mathrm{BSs}}=\frac{1}{3}\sum_{m=1}^{3}d_{BS,m}$, being $d_{BS,m}$ the distance between receiver and BS ordered by proximity. This definition is also used to identify those coverage areas with good positioning capabilities for trilateration techniques. This network density metric is depicted in Figure 1 for a grid of receiver positions over a coverage area. An average distance $d_{\mathrm{av},3\mathrm{BSs}}$ below 200 m (colored in blue) or above 500 m (colored in dark red) represents an urban or rural area, respectively, and the average distances between 200 and 500 m are considered suburban areas. This classification is here defined according to the typical ISD of small, macro and rural macro cells. This helps to easily associate each environment to a standard channel model for each type of cell deployment, such as the urban micro-cell (UMi), urban macro-cell (UMa) and rural macro-cell (RMa) channel models, which are used in Section 3 to simulate the cellular observables. Furthermore, an average height of buildings can be defined for each of these environments. Since the building height is the main limiting factor of the GNSS availability, our approach is to characterize the GNSS satellite visibility or elevation mask according to the density of BSs, as introduced in the following section.

### 2.2. GNSS Satellite Visibility

**Figure 2:**
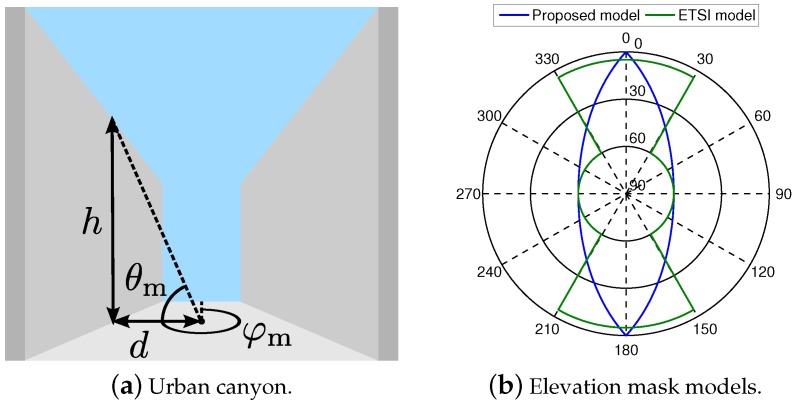
Definition of the urban canyon parameters and the sky plot of its elevation mask, according to ETSI TS 103.246-3 [13] and the proposed model.

The nominal conditions of satellite navigation systems are defined by an open sky scenario, because they are designed to operate only with line-of-sight (LoS) signals. Thus, the GNSS performance is severely degraded with a reduced satellite visibility and dense multipath, such as in urban and indoor environments. Indeed, the urban canyon is a typical example of harsh GNSS environment. As it is shown in Figure 2a, the buildings at both street sides block part of the sky, hence the number of visible GNSS satellites is reduced. Assuming a negligible diffraction contribution at the building edges, the height of the surrounding buildings determines the minimum elevation of a visible satellite, also known as the elevation mask $\theta_{m}$.

Several models can be found in the literature to define the elevation mask for straight street scenarios, such as those summarized in Table 1. The simplest GNSS visibility model is based on a constant elevation mask for a certain range of satellite azimuth values $\varphi_{m}$. As shown in Figure 2b, the elevation mask of the urban canyon scenario is set to 60° for a range of azimuths corresponding to the blocking buildings and to 5° for the rests of azimuths, according to the ETSI specification in [13]. However, this model is not accurate in terms of a representative building, because a constant elevation mask results in a building height that increases along the straight street (instead of being constant as expected). This can be circumvented with a generalized elevation mask model as in [20], where representative streets are modeled based on the street width, the building height and the receiver location within the street. Our approach is to simplify this generalized model for a receiver located in the middle of a symmetrical street, where only the aspect ratio of the street needs to be defined. As in [21], the elevation mask is then defined as function of the satellite azimuth $\varphi_{m}$ as

(1) $$\theta_{m}\left(\varphi_{m}\right)=\left|\mathrm{atan}\left(\frac{h}{d}\cdot \cos\left(\varphi_{m}\right)\right)\right|,$$

where *d* is the minimum distance between the receiver and the blocking building, and *h* is the height of the building. Indeed, the specific values of the distance to the building and its height do not strictly need to be defined, but their ratio can be obtained for a maximum elevation mask $\theta_{\max}$ at $\varphi_{m}=0$, as $h/d=\mathrm{atan}(\theta_{\max})$. In this sense, the proposed model can be compared with the standard model in [13] for a symmetrical infinite road and $\theta_{\max}=60^{\circ }$, as shown in Figure 2b. For the sake of simplicity, the elevation mask of the receiver antenna and a finite road length are not considered in this work, but these parameters can be easily adopted as boundaries of the proposed model.

**Figure 3:**
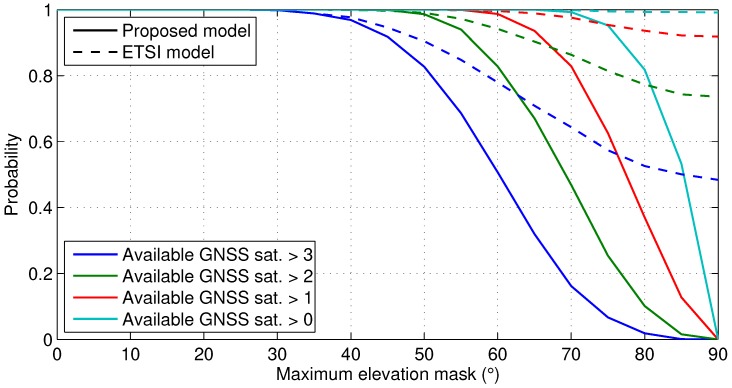
Probability of a minimum number of visible GNSS satellites per maximum elevation mask for the ETSI TS 103.246-3 [13] and the proposed models.

The impact of the standard and the proposed models can now be compared as function of the maximum elevation mask. Let us consider the elevation and azimuth of the GPS satellite orbits over one day in the north hemisphere. For instance, the GPS orbits are obtained from the public database of the ground reference station of Delft University of Technology with latitude 51°59′09.7″ N and longitude 4°23′14.9″ E on the 16 March 2016 at 00:00:01 UTC. Then, the GPS satellite visibility is computed by filtering the visible satellites for a certain elevation mask. The standard and the proposed models are used to define the elevation mask as function of the satellite azimuth. A symmetrical and straight street is considered, such as in Figure 2, and the street orientation is randomly distributed over 50,000 iterations at different epochs. The resulting visible satellites per epoch are used to compute the probability of observing at least one, two, three or four satellites for a maximum elevation mask, which is defined between 0° and 90°. As shown in Figure 3, the GPS constellation is able to provide full position availability (i.e., four or more visible satellites) for a maximum elevation mask of 30°. Thus, both models have the same impact for rural and suburban areas. However, in urban areas with a high density of buildings, the standard model is too optimistic, and it may fail to characterize the low satellite visibility. Thus, the proposed model is a generic and representative model, which can be characterized only with the maximum elevation mask.

**Figure 4:**
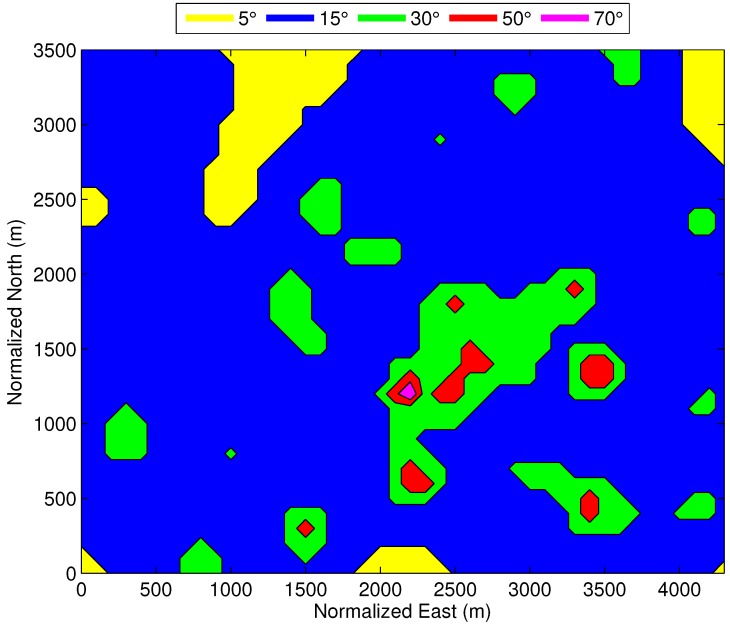
Elevation mask defined by the average distance to the three closest BSs of a representative cellular network deployment.

Let us now couple the cellular network density with the GNSS visibility, in order to define a generic hybrid scenario. As described in the previous section, the urban, suburban and rural areas are classified according to the density of BSs, by using the average distance metric $d_{\mathrm{av},3\mathrm{BSs}}$. The GNSS visibility is then determined with a set of maximum elevation masks for the different environments. An educated guess of these elevation masks is provided in Table 2, by considering the typical building height and street width of the corresponding scenario. Considering the representative cellular deployment of Figure 1, the resulting $d_{\mathrm{av},3\mathrm{BSs}}$ for the grid of receiver positions is used to define the GNSS visibility regions shown in Figure 4.

## 3. Characterization of the Observables

This section describes the methodology to characterize the GNSS and cellular observables. This methodology is aimed at reducing the complexity of the simulations, in order to ensure a clear reproducibility and to focus on the main impairments of each technology.

### 3.1. GNSS

The GNSS pseudoranges or code observables are mainly affected by satellite clock and orbit errors, atmospheric effects, multipath effects, the receiver noise and the receiver clock offset. A classical approach to correct the satellite clock, orbit and atmospheric errors is based on differential GNSS (DGNSS), which uses differential corrections from one or multiple GNSS reference stations located a few tens of km from the receiver [22]. In addition, precise corrections could also be obtained with a 5G network service. However, the receiver still needs to cope with the local errors due to multipath and receiver impairments. Due to the low bandwidth of the GNSS signals, i.e., the main lobe of the GPS L1 C/A signal is equal to 2.046 MHz, the multipath rays typically overlap with the LoS signal in the delay domain, resulting in a significant bias of the GNSS ranging observable. Therefore, the multipath-affected observables can be detected with advanced techniques, which can be based on the correlation asymmetry [23,24], on 3D building maps [25] or on multi-frequency signal-to-noise ratio (SNR) measurements [26] among others, and these biased observables are preferably discarded in the navigation solution. In this sense, the GNSS code observables are here modeled with only pseudoranges from visible satellites in LoS conditions, where differential or precise corrections are already applied. These ranging measurements are characterized by the effect of the receiver noise, being the clock offset an estimation parameter of the navigation solution. The receiver noise follows a Gaussian distribution with zero mean and a certain standard deviation $\sigma_{\mathrm{GNSS}}$. The minimum achievable standard deviation is limited by the Cramér-Rao bound (CRB), which is written as

(2) $$\begin{array}{cc} \sigma_{\mathrm{GNSS}} & =\sqrt{{\mathrm{CRB}}_{\mathrm{GNSS}}}=\sqrt{\frac{1}{8\pi^{2}\cdot T\cdot C/N_{0}\left(\theta \right)\cdot \int_{-F_{s}/2}^{F_{s}/2}f^{2}\cdot G_{s}\left(f\right)df}}, \end{array}$$

where *T* is the total integration time, $C/N_{0}\left(\theta \right)$ is the carrier-to-noise-density ratio of the GNSS signal as function of the satellite elevation θ, $F_{s}$ is the sampling frequency and $G_{s}\left(f\right)$ is the power spectral density of the GNSS signal. The total integration time is related with the equivalent bandwidth $B_{L}$ of the tracking loops as $T=1/(2B_{L})$. As usually considered in weighted-least square (WLS) algorithms [27], the quality of the GNSS signal can be modeled according to the satellite elevation as

(3) $$C/N_{0}\left(\theta \right)=\frac{{\left(C/N_{0}\right)}_{\mathrm{ref}}}{\sin^{2}\left(\theta \right)},$$

where ${\left(C/N_{0}\right)}_{\mathrm{ref}}$ is the reference $C/N_{0}$.

**Figure 5:**
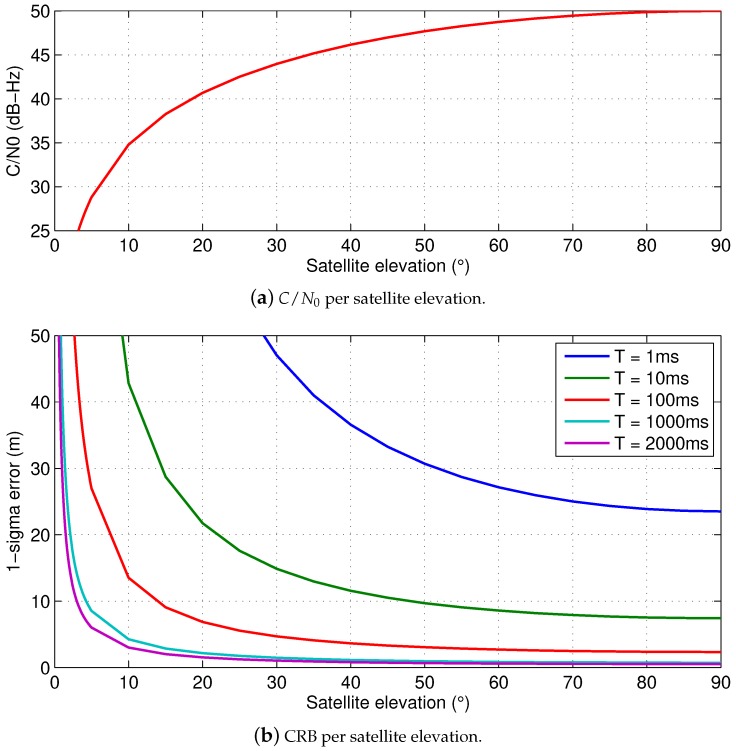
*C*/*N*_0_ and standard deviation of the GNSS observables as a function of the satellite elevation, by considering a GPS L1 C/A signal sampled at *F_s_* = 4 MHz.

Let us show an example of the achievable ranging accuracy by evaluating the CRB in (2) as a function of a satellite elevation θ, for the GPS L1 C/A signal and a receiver with a sampling frequency of 4 MHz, i.e., $F_{s}=4$ MHz. As it can be seen in Figure 5a for ${\left(C/N_{0}\right)}_{\mathrm{ref}}=50$ dB-Hz, the $C/N_{0}$ gradually decreases with the satellite elevation down to a level below 35 dB-Hz for satellite elevations below 10°. This simulated $C/N_{0}\left(\theta \right)$ is then used in (2) for different integration times. As it shown in Figure 5b, the ranging accuracy of the GNSS code observables is typically below 10 m for a delay tracking loop with a 5-Hz bandwidth, i.e., T=100 ms, which is a typical design value.

### 3.2. cmWave

The exploitation of cellular networks for positioning purposes can be based on opportunistic or dedicated approaches. Opportunistic positioning uses the cellular transmissions in the downlink without any interaction with the network operator. The main impairments of this approach are the inter-cell interference, multipath, receiver noise and lack of knowledge of the network, such as the precise position and time synchronization of the BSs. Most of these impairments can be avoided with dedicated positioning services, where the network operator dedicates spectrum resources, protocols and network equipment to determine the mobile location with downlink or uplink transmissions. For instance, the LTE standard specifies the positioning reference signal (PRS) within low-interference subframes and LTE positioning protocol (LPP), in order to perform downlink observed time-difference of arrival (OTDoA) positioning. In addition, the cellular network can increase the amount of consecutive positioning subframes, in order to compute ranging measurements over an extended integration time to cope with the noise effects. Therefore, the main source of ranging error in cellular networks is due to the effect of multipath, especially with the relative narrow bandwidth allocated at cmWave bands. In this sense, our characterization of the cmWave observables is based only on the multipath-induced error.

The ranging bias is here determined by the accuracy of a threshold-based time-delay estimator in a certain multipath channel. These estimators compute the cross-correlation function $R_{\mathrm{yx}}\left(\tau \right)$ between the received signal and the transmitted positioning pilots, and then they provide a time-delay estimate for the first peak above a certain threshold. Due to their low complexity, they are widely adopted in the literature. In addition, the performance of advanced multipath mitigation techniques is comparable to these threshold-based estimators for non-resolvable multipath and NLoS conditions. The time-delay estimation of the threshold-based approach is defined as

(4) $$\hat{\tau }=\min_{\tau_{\min}\leq \tau <\tau_{\max}}\left\{\tau \right\}\;s.t.\;\Lambda \left(\tau \right)\geq \Lambda_{\mathrm{thr}},$$

where the estimation range is bounded by $\tau_{\min}$ and $\tau_{\max}$, and the likelihood function is $\Lambda \left(\tau \right)={\left|R_{\mathrm{yx}}\left(\tau \right)\right|}^{2}$. Let us define the cross-correlation function in the absence of noise as

(5) $$\begin{array}{cc} R_{\mathrm{yx}}\left(\tau \right) & =R_{\mathrm{xx}}\left(\tau \right)*h\left(\tau \right), \end{array}$$

where $R_{\mathrm{xx}}\left(\tau \right)$ is the auto-correlation function, and $h\left(\tau \right)$ is the channel impulse response (CIR). Since the 5G physical layer is based on the orthogonal frequency division multiplexing (OFDM), the auto-correlation of the cmWave signal is

(6) $$R_{\mathrm{xx}}\left(\tau \right)=\sum_{n\in N}e^{-j\frac{2\pi n\tau }{N}},$$

where *n* is the pilot index within the set of subcarriers N from a total of *N* subcarriers. The CIR is modeled as

(7) $$\begin{array}{cc} h\left(\tau \right) & =\sum_{k=0}^{L-1}h_{k}\cdot \delta \left(\tau -\tau_{k}\right), \end{array}$$

where $h_{k}$ is the channel coefficient and $\tau_{k}$ is the tap delay for the *k*-th tap from a total of *L* multipath taps.

The 5G cellular standard specifies in [16] geometry-based stochastic channels, where the delay distribution of the multipath rays mainly depends on the propagation scenario, the LoS conditions, and the carrier frequency. These channel models are an extension (to mmWave transmissions) of the WINNER II channel models in [28], where the main differences are based on the scaling of the channel model parameters as a function of the carrier frequency. Since this section focuses on cmWave scenarios, the UMi, UMa and RMa scenarios of the WINNER II models are considered for the sake of simplicity. These models are based on tabulated large-scale parameters, such as the delay spread, for each propagation scenario, and the probability of LoS is defined as function of the distance between mobile and BS. Considering these models, our approach is to characterize the ranging error or estimation bias, i.e., $b\left(\hat{\tau }\right)=E\left[\hat{\tau }\right]-\tau$, independently of the distance between mobile and BS. This implies to compute the cumulative density function (CDF) of the ranging error for multipath channel realizations with LoS and for realizations with NLoS, considering the threshold-based estimator for a certain signal bandwidth and propagation scenario. Then, the LoS conditions are computed according to the distance between mobile and BSs $d_{\mathrm{BS}}$, as in [29], and the corresponding CDF for LoS or NLoS conditions is randomly evaluated to generate ranging errors. These errors are finally used with the distance to the BS to compute the cmWave ToA observables, i.e., $\hat{d}=d_{\mathrm{BS}}+c\cdot b\left(\hat{\tau }\right)$, where *c* is the speed of light. This approach reduces the computational burden of the generation of observables, while they are representative of the standard channel models.

**Figure 6:**
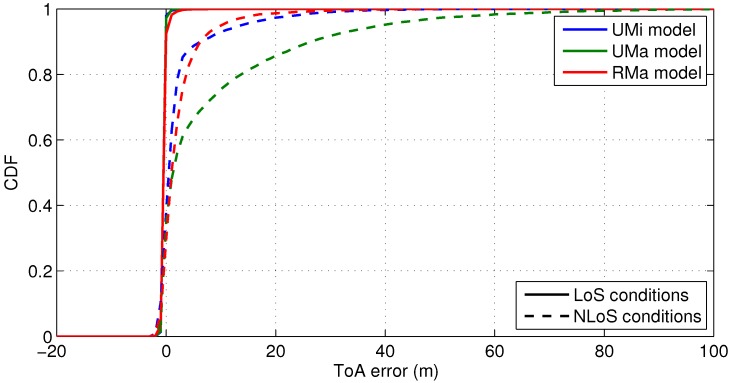
CDF of the ranging errors with a cmWave signal of 90-MHz bandwidth for WINNER channel models in LoS and NLoS conditions.

As an example, let us consider an OFDM cmWave signal, i.e., at a carrier frequency of 2 GHz, with a signal bandwidth of 90 MHz, which is formed by 6000 contiguous pilot subcarriers and a subcarrier spacing of 15 kHz. The range of the time-delay estimation is set to $\tau \in \left[-20,100\right]/c$ and the likelihood threshold is defined as $\Lambda_{\mathrm{thr}}≃\max\left(\Lambda \left(\tau \right)\right)/4$. The resulting CDF of the threshold-based ranging errors is shown in Figure 6 for LoS and NLoS conditions over UMi, UMa and RMa scenarios. As it can be seen, the NLoS ranging bias is above 10 m for the 95% of the cases, while the LoS ranging error is below one meter.

### 3.3. mmWave

With 5G technology and more specifically with the combination of mmWave and beamforming, new solutions for cellular network-based positioning can be developed [8]. An attractive and promising approach is the single-BS solution described in [30], which leverages angle-of-arrival, angle-of-departure and ranging estimations to provide accurate user equipment (UE) position and rotation also in mixed LoS and NLoS channel conditions.

The mathematical model underpinning this approach relies on: (a) the channel sparsity at the high carrier frequency [31] and (b) the geometric relationship between channel parameters and UE and scatter (reflection point) locations, i.e., channel impulse response is given as,

(8) $$H\left(t,\tau ,\phi^{t},\theta^{t},\phi^{r},\theta^{r},h\right)=\sum_{k=0}^{K}h_{k}a_{R}\left(\theta_{k}^{r},\phi_{k}^{r}\right)a_{T}^{H}\left(\theta_{k}^{t},\phi_{k}^{t}\right)\delta \left(t-\tau_{k}\right),$$

where $a_{R}\left(\phi ,\theta \right)$ and $a_{T}\left(\phi ,\theta \right)$ are the steering vectors of the receive and transmitting antennas, *k* indicates the index of the *k*-th channel path (with k=0 and k>0 for the LoS and NLoS paths), $h,\tau ,\phi^{t},\theta^{t},\phi^{r},\theta^{r}$ are the vectors of channel coefficients, time-delays, azimuths and elevations of the AoA and AoD of the K+1 paths with $\tau_{k}$, $\phi_{k}^{r},\theta_{k}^{r}$ and $\phi_{k}^{t},\theta_{k}^{t}$ given by

(9) $$\tau_{0}={∥p-q∥}_{2}/c,$$

(10) $$\phi_{0}^{r}=\arctan(\left(p_{y}-q_{y}\right)/\left(p_{x}-q_{x}\right)),$$

(11) $$\theta_{0}^{r}=\arctan(\left(p_{z}-q_{z}\right)/∥p-q{∥}_{xy}),$$

(12) $$\phi_{0}^{t}=\arctan(\left(q_{y}-p_{y}\right)/\left(q_{x}-p_{x}\right))-\alpha ,$$

(13) $$\theta_{0}^{t}=\arctan(\left(q_{z}-p_{z}\right)/∥q-p{∥}_{xy})-\beta ,$$

(14) $$\tau_{k}=\tau_{k:1}+\tau_{k:2}=(∥q-s_{k}{∥}_{2}+∥s_{k}-p{∥}_{2})/c,\;\forall \;k>0$$

(15) $$\phi_{k}^{r}=\arctan(\left(s_{k:y}-q_{y}\right)/\left(s_{k:x}-q_{x}\right)),\;\forall \;k>0$$

(16) $$\theta_{k}^{r}=\arctan(\left(s_{k:z}-q_{z}\right)/∥s_{k}-q{∥}_{xy}),\;\forall \;k>0$$

(17) $$\phi_{k}^{t}=\arctan(\left(s_{k:y}-p_{y}\right)/\left(s_{k:x}-p_{x}\right))-\alpha ,\;\forall \;k>0$$

(18) $$\theta_{k}^{t}=\arctan(\left(s_{k:z}-p_{z}\right)/∥s_{k}-p{∥}_{xy})-\beta ,\;\forall \;k>0$$

being $p≜{\left[p_{x},p_{y},p_{z}\right]}^{T}$, $q≜{\left[q_{x},q_{y},q_{z}\right]}^{T}$, $s≜{\left[s_{k:x},s_{k:y},s_{k:z}\right]}^{T}$ the coordinates of the UE, BS and *k*-th scatter, respectively, α and β the azimuth and elevation offset of the UE with respect to the BS orientation, ${∥\cdot ∥}_{2}$ denoting the Euclidean norm, and ${∥\cdot ∥}_{xy}$ the Euclidean norm of xy components of the three-dimensional (3D) vector.

Relying on this model, physical channel parameters and, subsequently, location and orientation of the UE can be estimated from the received reference signal. The signal model is valid for both uplink and downlink transmission. More specifically, for an OFDM transmission, the time-domain expression of the received signal is given by

(19) $$Y\left(t\right)=\sqrt{\frac{P_{t}}{N}}\sum_{k=0}^{K}\sum_{i=1}^{N}h_{k}W^{H}a_{R}\left(\theta_{k}^{r},\phi_{k}^{r}\right)a_{T}^{H}\left(\theta_{k}^{t},\phi_{k}^{t}\right)Fs_{i}e^{j2\pi n_{i}\Delta_{f}\left(t-\tau_{k}\right)}+W^{H}n\left(t\right),$$

where $P_{t}$ is the OFDM transmission power, n(t) is the white noise, *N* is the number of pilot subcarriers, $n_{i}$ is the *i*-th pilot subcarrier index, $s_{i}\in C^{N_{r}}$ is the pilot vector used on the *i*-th subcarrier and $W\in C^{M_{r}\times M}$, $F\in C^{N_{r}\times N}$ are the receiver and transmitter beamformer matrix, respectively.

As shown in Appendix A the fundamental limits for channel parameters estimation can be derived in closed-form. The analysis is carried on for a simplified scenario with only LoS and uniform linear array antennas.The following conclusions can be devised: (i) ranging performance (Equation (A17)) depends on the transmit and beamforming gain, the subcarrier spacing and the allocation to the pilot subcarriers, i.e., higher index, higher ranging information; (ii) angle information (Equations (A18) and (A19)) depends on the beamforming gain as well as on the sensitivity of the beampattern to the angle variations (derivative term) and, (iii) the information on the LoS AoA and AoD are coupled (Equation (A22)).

**Figure 7:**
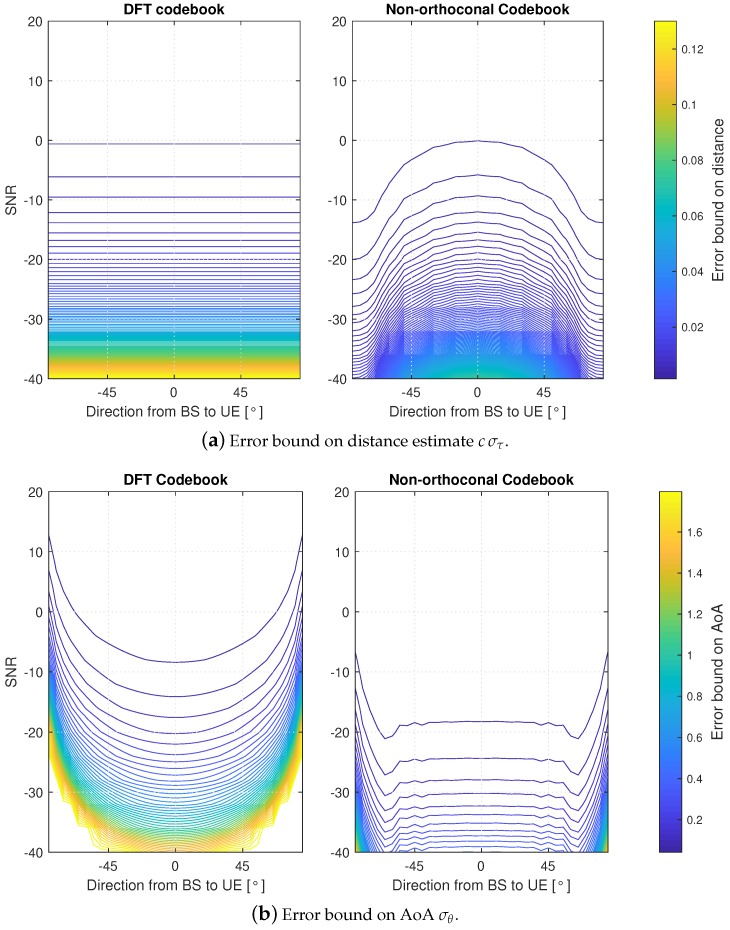
Error bounds on estimated mmWave channel parameters.

In Figure 7a,b a contour plot of the achievable ranging and AoA estimation error are shown. The curves are drawn as a function of the location of the UE (LoS angle) as well as the SNR computed without the beamforming gain. The achievable estimation errors are investigated with two different codebooks, namely the orthogonal Discrete Fourier Transform (DFT) codebook and the non-orthogonal codebook. Size of the DFT codebook (i.e., number of beams) is N=16 while non-orthogonal codebook uses 32 beams. The simulation uses subcarrier spacing $\delta_{f}=60$ kHz and number of subcarriers N=2048, which all are used in estimation. Simulations sweeps the range [−π,π] on angular domain, and $E_{s}/N_{0}$ range from −40 to 20 dB.

In Figure 7a, we note that the error bound on distance estimation with DFT codebook does not change within the full angular domain. This is due to the orthogonal property of the codebook and the fact that cumulative beamforming gain is constant once the whole codebook is scanned. Oppositely, with non-orthogonal beams, the performance varies with the angle and a lower error can be achieved on the edges of the angular range. Also, the ranging error with non-orthogonal codebook is lower with respect to that one obtained with DFT-based codebook.

The reason is two-fold. First, if the channel-ray is aligned with the direction of maximum gain of a beam, also side-lobes of any other beam in the non-orthogonal codebook can provide a useful gain. Thus, the Fisher Information Matrix (FIM) component corresponding to ranging information is higher and, subsequently, the ranging error is lower. Second, near the fire-end direction, more than one beam in the non-orthogonal codebook provides non-zero gain. Therefore, also in this case, ranging information can benefit of a cumulative higher transmit and receive beamforming gain.

Next, we consider the angular error shown in Figure 7b. It can be noticed that not only bearing error is generally better with non-orthogonal codebook, but also a wider range of directions can be estimated more accurately and with similar level of precision. Reasons are similar as above.

In light of the above, the error model on the observables shall include multiple factors: (i) beamforming codebook, (ii) SNR without array gain, i.e., $E_{s}/N_{0}$, (iii) subcarrier spacing and, (iv) number of subcarrier pilots. Hereafter, we shall denote the observables as follows

(20) $$\begin{array}{ccc} {\tilde{d}}_{k} & = & c\tau_{k}+n_{d}, \end{array}$$

(21) $$\begin{array}{ccc} {\tilde{\theta }}_{k}^{t} & = & \theta_{k}^{t}+n_{\theta_{t}}, \end{array}$$

(22) $$\begin{array}{ccc} {\tilde{\phi }}_{k}^{t} & = & \phi_{k}^{t}+n_{\phi_{t}}, \end{array}$$

(23) $$\begin{array}{ccc} {\tilde{\theta }}_{k}^{r} & = & \theta_{k}^{r}+n_{\theta_{r}}, \end{array}$$

(24) $$\begin{array}{ccc} {\tilde{\phi }}_{k}^{r} & = & \phi_{k}^{r}+n_{\phi_{r}}, \end{array}$$

where noise terms $n_{d},n_{\theta_{t}},n_{\phi_{t}},n_{\theta_{r}}$ and $n_{\phi_{r}}$ are error terms computed from the inverse of the FIM.

## 4. Position Solution

This section describes the positioning algorithm considered for the hybridisation of GNSS pseudoranges with cmWave observables and for the use of mmWave observables. The unknown parameters for both cases are the 3D mobile position and the receiver clock offset.

### 4.1. Tightly Coupled GNSS and cmWave Cellular Positioning

**Figure 8:**
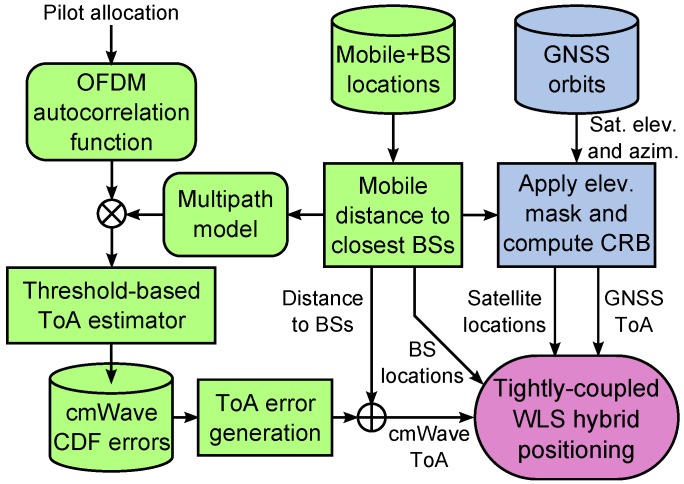
Simulation methodology of tightly coupled WLS hybrid GNSS and cmWave positioning.

The general architecture of the tightly coupled hybridisation of GNSS and cmWave observables is shown in Figure 8. First, the network deployment and the mobile locations are used to define the outdoor urban, suburban and rural scenarios. As described in Section 2, the average distance from the mobile to the three closest BSs is used to characterize the network density and the satellite elevation mask. Then, the statistics of the cmWave observables are generated according to the channel models for each environment. The GNSS and cmWave ToA observables are finally simulated by considering the mobile location and by evaluating the distribution of the GNSS and cmWave ranging errors. These ToA observables are used to compute the mobile position with a WLS algorithm.

Let us define the *m*-th ranging observable as

(25) $$\begin{array}{c} {\hat{\rho }}_{m}=c\cdot {\hat{\tau }}_{m}=∥x_{m}-x∥+c\cdot \delta t+e_{m}, \end{array}$$

where ${\hat{\tau }}_{m}$ is the time-delay estimated from the *m*-th transmitter, which can be a GNSS satellite or cmWave BS, $x_{m}={\left[x_{m},y_{m},z_{m}\right]}^{T}$ and $x={\left[x,y,z\right]}^{T}$ are the locations of the transmitter and mobile receiver, respectively, δt is the receiver clock offset with respect to the reference GNSS time, and $e_{m}$ is the ranging error. Given the known location of the transmitters, the unknown parameters are $\theta ={\left[x,y,z,\delta t\right]}^{T}$. The WLS classical solution of this trilateration problem is formulated as the nonlinear least squares (NLS) minimization

(26) $$\begin{array}{c} \hat{\theta }=\left[\begin{array}{c} \hat{x}\\ \hat{y}\\ \hat{z}\\ \hat{\delta t} \end{array}\right]=\arg\min_{\theta }\left\{∥\rho \left(\theta \right)-\hat{\rho }{∥}_{W}^{2}\right\}, \end{array}$$

where $\rho \left(\theta \right)={\left[\rho_{1}\left(\theta \right),\rho_{2}\left(\theta \right),\cdots ,\rho_{M}\left(\theta \right)\right]}^{T}$, being $\rho_{m}\left(\theta \right)=∥x_{m}-x∥+c\cdot \delta t$, and $\hat{\rho }={\left[{\hat{\rho }}_{1},{\hat{\rho }}_{2},\cdots ,{\hat{\rho }}_{M}\right]}^{T}$ for a total number of transmitters *M*. The *M* transmitters used for hybrid positioning is the sum of the number of visible GNSS satellites $M_{\mathrm{sat}}$ and the number of strongest cmWave BSs $M_{\mathrm{BS}}$, i.e., $M=M_{\mathrm{sat}}+M_{\mathrm{BS}}$. Our WLS implementation is based on the well-known iterative Gauss-Newton (GN) method. Then, the GN solution at the *ℓ*-th iteration is defined as

(27) $${\hat{\theta }}_{\ell }={\hat{\theta }}_{\ell -1}+{\left(G^{T}W^{-1}G\right)}^{-1}G^{T}W^{-1}\left(\rho \left({\hat{\theta }}_{\ell -1}\right)-\hat{\rho }\right),$$

where the Jacobian matrix or geometry matrix of $\rho (\hat{\theta })$ is a M×4 matrix defined as

(28) $${\left[G\right]}_{m,1:4}=\left[\begin{array}{cccc} \frac{x_{m}-\hat{x}}{\rho_{m}\left(\hat{\theta }\right)} & \frac{y_{m}-\hat{y}}{\rho_{m}\left(\hat{\theta }\right)} & \frac{z_{m}-\hat{z}}{\rho_{m}\left(\hat{\theta }\right)} & -1 \end{array}\right],$$

and W is the diagonal weighting matrix. The weighting coefficients of the GNSS observables are defined with (2) as $w_{\mathrm{sat}}=c\cdot {\left[\sigma_{\mathrm{sat},1},\sigma_{\mathrm{sat},2},\cdots ,\sigma_{\mathrm{sat},M_{\mathrm{sat}}}\right]}^{T}$, while the cmWave weighting coefficients are just a unitary vector of $M_{\mathrm{BS}}$ BSs, i.e., $w_{\mathrm{BS}}=1_{M_{\mathrm{BS}}\times 1}$. Thus, the weighting matrix is written as

(29) $$W=\left[\begin{array}{cc} \mathrm{diag}\left(w_{\mathrm{sat}}\right) & 0_{M_{\mathrm{sat}}\times M_{\mathrm{BS}}}\\ 0_{M_{\mathrm{BS}}\times M_{\mathrm{sat}}} & \mathrm{diag}\left(w_{\mathrm{BS}}\right) \end{array}\right].$$

The cmWave observables are equally weighted because there is no prior knowledge of the LoS or NLoS conditions. In case of perfect knowledge of the propagation conditions, the weighting coefficients of the cmWave NLoS observables are set to zero, in order to only exploit the LoS observables with weights equal to one.

The location problem can be evaluated in terms of position and timing with the geometric dilution of precision (GDOP), by using the true receiver position. This metric, which predicts the precision of the position solution, is here defined for the two-dimensional (2D) case as

(30) $${\mathrm{GDOP}}_{2D}=\sqrt{\mathrm{tr}\left\{{\left(G_{2D}^{T}G_{2D}\right)}^{-1}\right\}},$$

where ${\left[G_{2D}\right]}_{m,1:3}=\left[\begin{array}{ccc} \frac{x_{m}-x}{\sqrt{{\left(x_{m}-x\right)}^{2}+{\left(y_{m}-y\right)}^{2}}} & \frac{y_{m}-y}{\sqrt{{\left(x_{m}-x\right)}^{2}+{\left(y_{m}-y\right)}^{2}}} & -1 \end{array}\right]$ is the reference 2D geometry matrix, and for the 3D case as

(31) $${\mathrm{GDOP}}_{3D}=\sqrt{\mathrm{tr}\left\{{\left(G_{3D}^{T}G_{3D}\right)}^{-1}\right\}},$$

where ${\left[G_{3D}\right]}_{m,1:4}=\left[\begin{array}{cccc} \frac{x_{m}-x}{∥x_{m}-x∥} & \frac{y_{m}-y}{∥x_{m}-x∥} & \frac{z_{m}-z}{∥x_{m}-x∥} & -1 \end{array}\right]$ is the reference 3D geometry matrix. When the GDOP is above 6, there is a deficient geometry between transmitters and receiver that leads to position outages, while a GDOP value below 2 is excellent to achieve a precise solution. The positioning performance is then assessed with the 2D position error defined as $\varepsilon_{2D}=\sqrt{{\left(\hat{x}-x\right)}^{2}+{\left(\hat{y}-y\right)}^{2}}$, and the 3D position error as $\varepsilon_{3D}=∥\hat{x}-x∥$.

### 4.2. Stand-Alone mmWave Positioning

The position and rotation estimation for a 5G mmWave system is based on the following non-linear-weighted-least-squares (NWLS) optimization problem.

(32) $$\left[\hat{p},\hat{\alpha },\hat{\beta }\right]=\underset{p,\hat{\alpha },\hat{\beta }}{\mathrm{argmin}}\;{∥\bar{\eta }-\bar{f}{\left(p,\alpha ,\beta \right)}^{T}∥}_{\Xi },$$

where the observation vector $\bar{\eta }≜{\left[\eta_{1}^{T},\cdots ,\eta_{N_{a}}^{T}\right]}^{T}$, and Ξ is the weighing matrix including estimates of the error variances of the observables, $\bar{f}\left(p,\alpha ,\beta \right)≜{\left[f_{1}^{T}\left(p,\alpha ,\beta \right),\cdots ,f_{N_{a}}^{T}\left(p,\alpha ,\beta \right)\right]}^{T}$ and $f_{i}\left(\cdot \right)$ is the vector function including the LoS and NLoS relationships between the user’s location-rotation information and the channel parameters corresponding to the link between the *i*-th base station and the user, i.e., Equations (9)–(18) $\forall i=1,\ldots ,N_{a}$.

In the simple case of LoS (or dominant LoS) channel condition, the above optimization includes solely the LoS equations yielding to a non-linear system of $N_{a}\times 5$ equations. The solution is sought with a standard iterative algorithm (e.g., the Levenberg-Marquardt), which can be initialized with

(33) $${\hat{p}}_{0}=\frac{1}{N_{a}}\sum_{i=1}^{N_{a}}{\bar{p}}^{i},$$

(34) $${\left[{\hat{\alpha }}_{0},{\hat{\beta }}_{0}\right]}^{T}=\frac{1}{N_{a}}\sum_{i=1}^{N_{a}}{\left[{\bar{\alpha }}^{i},{\bar{\beta }}^{i}\right]}^{T},$$

and defined as

(35) $$\begin{array}{cc} {\bar{p}}^{i}= & q^{i}+{\tilde{d}}_{0}^{i}\left[\begin{array}{c} \cos{\tilde{\theta }}_{0}^{r,i}\cos{\tilde{\phi }}_{0}^{r,i}\\ \cos{\tilde{\theta }}_{0}^{r,i}\sin{\tilde{\phi }}_{0}^{r,i}\\ \sin{\tilde{\theta }}_{0}^{r,i} \end{array}\right], \end{array}$$

(36) $$\begin{array}{cc} {\bar{\alpha }}^{i}= & \pi +{\tilde{\phi }}_{0}^{t,i}-{\tilde{\phi }}_{0}^{r,i}, \end{array}$$

(37) $$\begin{array}{cc} {\bar{\beta }}^{i}= & {\tilde{\theta }}_{0}^{r,i}-{\tilde{\theta }}_{0}^{t,i}, \end{array}$$

where the *i* superscript indicates a measurement or estimation respect to the *i*-th base station.

## 5. Performance Results

This section discusses the localization improvements of GNSS based on the use of cmWave or mmWave cellular networks. The simulation methodology is based on the definition of the outdoor urban, suburban and rural scenarios in Section 2, the characterization of GNSS, cmWave and mmWave observables in Section 3, where differential or precise corrections are applied to the GNSS code observables, and the positioning algorithms described in Section 4. The performance results are mainly obtained in terms of position accuracy and availability.

The representative network deployment shown in Figure 1 is used to determine the satellite visibility. The mobile location is defined with a rectangular grid of points with a resolution of 100 m over the coverage area of 4.35 km by 3.6 km, which results in 1584 different mobile positions. The urban areas are represented by 14.8% of the positions, i.e., 13.4%, 1.3% and 0.1% with 30°, 50° and 70° of elevation mask, respectively. The suburban scenarios are widely characterized with a 79.2% of the positions, while the rural scenarios is only shown in the 6% of the positions. The GPS constellation is only considered in order to assess nominal GNSS conditions. Thus, the use of multiple constellations is left for future work. For each mobile position, the positioning performance is evaluated over 200 iterations for the case of GPS and cmWave observables. Considering the same configuration as in the example of Figure 6, the cmWave observables are obtained with a threshold-based ToA estimator by using a 90-MHz signal bandwidth and a time-delay estimation range defined as $\tau \in \left[-20,100\right]/c$. The LoS propagation conditions of the cmWave signals depend on the distance to the BS, by following the UMi, UMa and RMa models in [29]. Depending on the LoS conditions, the cmWave observables are obtained from the pre-computed distribution of the LoS or NLoS ranging bias. This realistic scenario helps to identify the benefits achieved with the hybridisation between GNSS and cmWave signals. The use of the mmWave technology is only studied for urban scenarios with LoS-dominant connectivity. Also, the case with multiple (up-to three) BSs for a dense deployment scenario of mmWave access points is considered. Finally, BS and UE are considered at same height, thus elevation of AoD and AoA are not considered in the simulation model.

### 5.1. Suburban and Rural Scenarios

**Figure 9:**
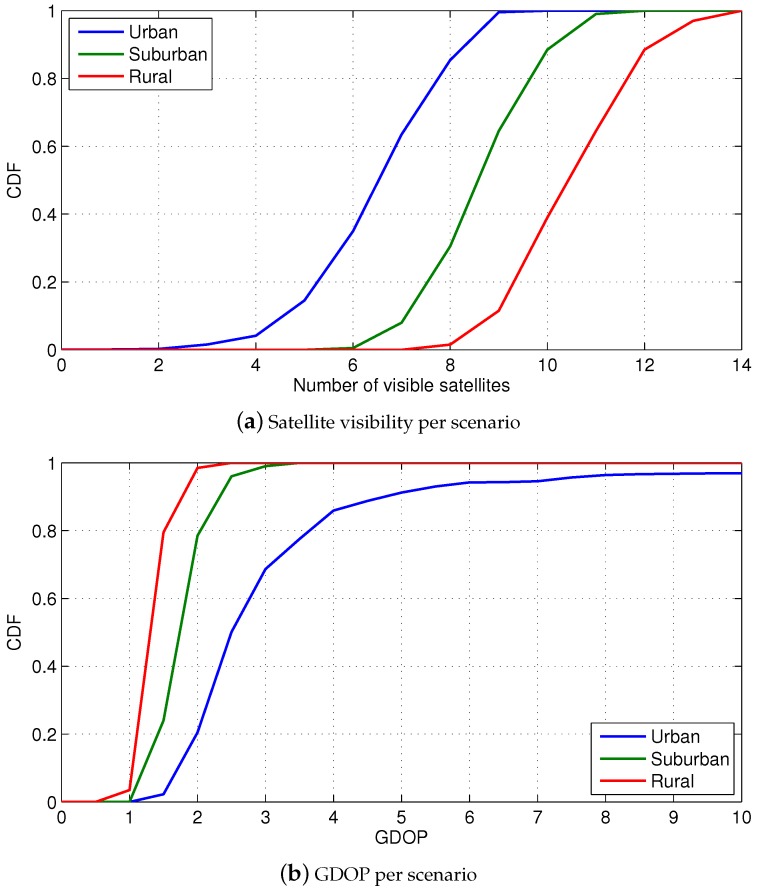
CDF of the GPS satellite visibility and GDOP per scenario.

**Figure 10:**
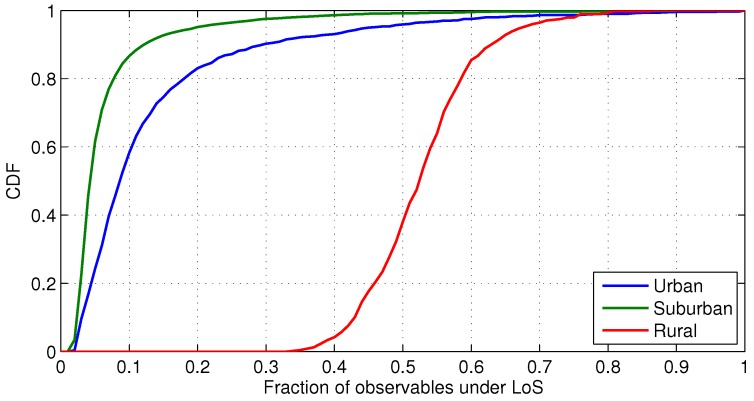
CDF of the fraction of cmWave observables under LoS conditions per scenario.

The dominant positioning technology in suburban and rural scenarios is GNSS due to the favourable visibility conditions. As described in Section 2, the low building height results in an elevation mask below 30°. The characterization of the suburban and rural scenario indicates that at least ten GPS satellites are in view on the 90% of the epochs of a day, as shown in Figure 9a. In contrast, there is a significant multipath effect on the cmWave communication channel over these environments. The suburban scenario is especially severe due to the NLoS conditions, according to the WINNER-based UMa model of the LoS probability. As described in Section 3, these LoS probability models are used in the simulation, resulting in the CDF of the fraction of observables under LoS conditions shown in Figure 10. This result indicates the severe conditions expected in cmWave propagation channels.

**Figure 11:**
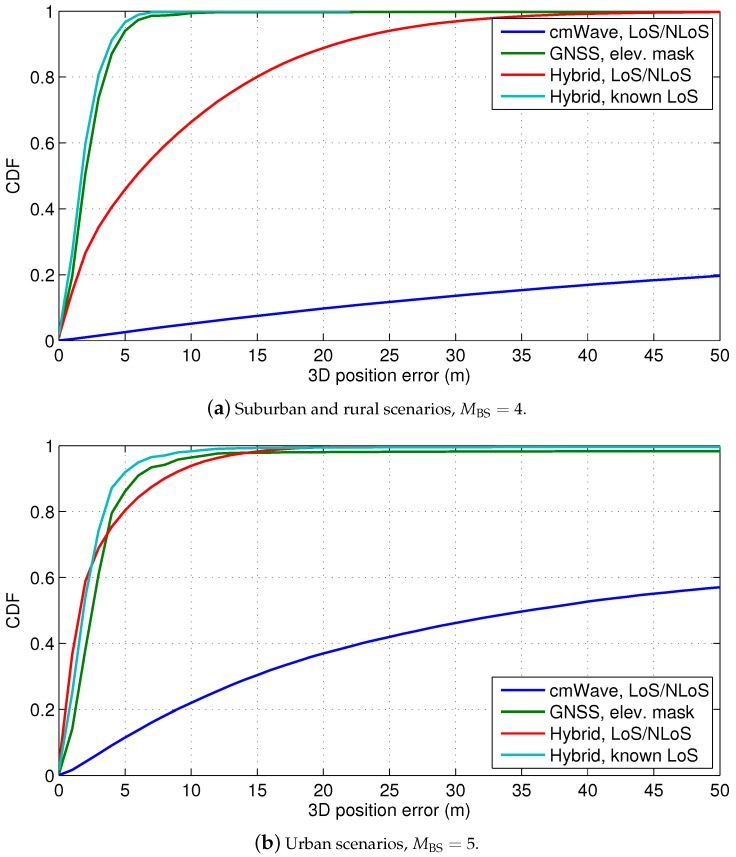
CDF of the 3D position error with hybrid GNSS and cmWave positioning in urban, suburban and rural scenarios.

The good GNSS visibility helps to achieve a good location geometry. As it can be seen in Figure 9b, the visible GPS constellation achieves a GDOP below 3 in most of the occasions. As a result, the GNSS position errors are around 5 m on the 95% of the cases, as shown in Figure 11a. Thus, GNSS (with differential or precise corrections) achieves high-accuracy positioning in suburban and rural areas, as expected.

The low density of the cmWave network in these scenarios results in a deficient horizontal location geometry. Furthermore, the high distance between the mobile and the BSs further degrades the vertical dilution of precision. Considering these scenarios, the CDF of the 2D position error and GDOP, i.e., $\varepsilon_{2D}$ and ${\mathrm{GDOP}}_{2D}$, is computed with cmWave ToA positioning. As it can be seen in Table 3, there is a high GDOP with values above ten, which results in a severe performance degradation. Considering only $M_{\mathrm{BS}}=3$, the 2D cmWave position errors are around 100 m on the 95% of the cases. The use of more BSs helps to improve the positioning performance, but the position errors are still higher than 25 m and 50 m in rural and suburban environments, respectively. These results show the severe performance degradation due to the lack of BSs, e.g., when using only $M_{\mathrm{BS}}=3$. This poor positioning performance is mainly due to the harsh NLoS conditions and the bad geometry of the BSs. Thus, the proposed characterization of cmWave observables, which is only based on the multipath impact, is valid for the evaluation of the achievable snapshot positioning performance, due to the dominant NLoS ranging bias.

In case of lack of knowledge of the LoS conditions, the hybridisation of GNSS and cmWave ToA observables does not improve the GNSS accuracy, due to the significant NLoS ranging bias of the cmWave observables. As shown in Figure 11a and in Table 4, the 3D hybrid position error is indeed around 25 m higher than with only GNSS observables. This is because the cmWave observables in NLoS conditions corrupt the position estimation. Thus, the NLoS detection is required to exploit the accurate cmWave observables in LoS conditions. The prior information of NLoS conditions, such as with a NLoS detector, is considered in the weighting matrix of the WLS algorithm. As a result, the 3D hybrid position error slightly outperforms the GNSS performance. Therefore, the analysis of the LoS conditions, such as based on the CIR, is very important to achieve the best positioning accuracy. In this sense, standardization initiatives, such as multipath reporting in LTE enhancements [32], is of interest for high-accuracy localization.

### 5.2. Urban Scenarios with cmWave Deployments

The urban scenarios are characterized by different building profiles with elevation masks above 30°, as described in Section 2. However, the areas with high elevation masks, i.e., equal or above 50°, only represent 1.4% of the total number of positions, while moderate elevation masks of 30° are observed in the 13.4% of the cases. Thus, the GNSS urban visibility is only reduced to around 8 GPS satellites in view, as shown in Figure 9a. In addition, there is also a slight degradation on the GDOP of the GNSS solution. This degradation introduces a reduction of 1% on the probability of GNSS position fixes. As it shown in Figure 11, the use of cmWave observables helps to achieve the full position availability in urban scenarios. However, as discussed in the previous section, the cmWave observables in NLoS conditions are still the dominant error of the hybrid position solution. In this sense, the exploitation of wideband cmWave signals for precise snapshot localization is limited by the harsh urban propagation conditions. Nonetheless, the knowledge of the NLoS conditions and the removal of NLoS observables improves the hybrid position accuracy, i.e., to 6 m on the 95% of cases. This improvement is obtained at the expense of a 0.5% of occasions without position fixes, due to the existence of only cmWave NLoS observables and lack of GNSS observables. Thus, advanced weighting techniques need to be used in future work to cope with this reduction on the hybrid positioning availability, in order to weight both cmWave NLoS and GNSS observables.

### 5.3. Urban Scenarios with mmWave Deployments

In this study we consider a typical urban scenario mmWave radio access (up-to three equispaced BSs), deployed, for instance, at border of a circle with radius d=50 m or d=100 m. The UE location (in 2D) is selected randomly such that ${∥q∥}_{2}<d$. The rotation is also a random variable with uniform distribution α∈{−π,π}. Both at the UE and at each BS, a uniform linear array with N=8 and M=16 antenna elements are considered, respectively. The OFDM parameters for pilot transmissions are same as in Section 3.3. LoS is always assumed dominant.

Positioning is performed with a single base station (1 BS with LoS link) and multi-connectivity (3 BSs with LoS links) approach. The method is described in Section 4.2. Furthermore, both a DFT-based codebook and non-orthogonal codebook are considered to evaluate the impact of the beam scanning strategy (see Section 3.3) on the localization accuracy.

**Figure 12:**
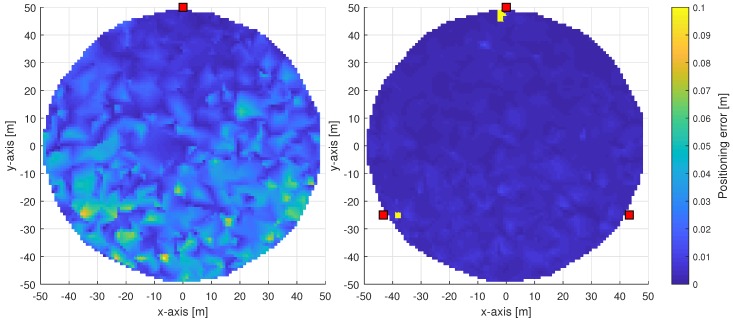
Urban scenarios (<50 m) mmWave NLS positioning using non-orthogonal codebook and LoS link available from one (**left side**) or three (**right side**) base stations.

**Figure 13:**
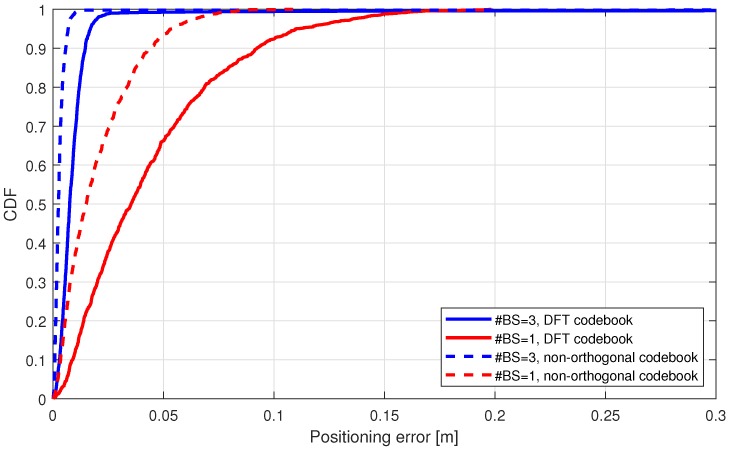
Urban scenarios (<50 m) mmWave NLS positioning with LoS link from one or three base stations.

First, we consider the scenario with 50 m radius and in Figure 12 we show the spatial distribution of the localization error with 1 or 3 connected BSs. As expected, the multi-connectivity approach outperforms the single-BS case in terms of localization accuracy and distribution of the low-error. This advantage can be better evaluated from Figure 13, where we can notice that in 90% of cases UE is located within <0.02 m accuracy, while in case of single-BS, the available accuracy is within <0.10 m. Also the distribution of the localization error is generally more smooth with the 3 BS scenario. In fact, with single BS scenario higher errors occur as the distance between UE and BS is greater at the bottom of the circular area. In the 3-BSs scenario, nonetheless, slight errors occur at the bottom of the circular area. This is due to the orientation of antennas on BSs, which are aligned horizontally so that the main lobe is directed along the *x*-axis.

In Figure 13 we also compare two different codebooks for beamsearch strategy-based. It is shown that non-orthogonal beams approximately provide 50% improvement over the DFT-based codebook, for instance, looking the CDF function of 90% accuracy.

**Figure 14:**
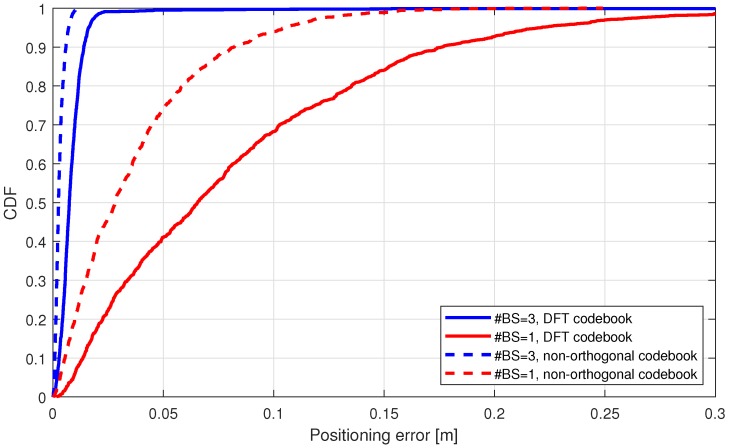
Urban scenarios (<100 m) mmWave NLS positioning with LoS link from one or three base stations.

Finally, we consider the scenario with 100 m radius. As in the study of the 50 m case, Figure 14 show the localization error of the cumulative distribution for 100 m case. Looking at the performance on 90% CDF point we note that the localization error on the single-BS scenario grows, e.g., in DFT codebook case, the CDF of 90% is achieved at 0.09 m within <50 m scenario and at 0.17 m within <100 m scenario. Oppositely, in the 3-BSs scenario, the localization error is very close in both scenarios (difference approx. only 0.01 m), in both cases, with DFT and with non-orthogonal codebook.

## 6. Conclusions

This paper presents a representative methodology to evaluate the hybridisation of fifth generation (5G) cellular observables with Global Navigation Satellite Systems (GNSS). This methodology is based on a novel scenario definition, where the GNSS visibility masks are related to the network density. Based on a realistic network deployment and standard models, GNSS, centimetre-wave (cmWave) and millimetre-wave (mmWave) observables are characterized according to the main scenario parameters, such as the satellite elevation or the multipath effect. Using a snapshot localization approach, the hybrid fusion of GNSS and cmWave observables is first evaluated with the proposed methodology. The simulation results indicate the limited positioning improvement of cmWave observables, due to the dominant non-line-of-sight (NLoS) propagation conditions. Therefore, the knowledge of these NLoS conditions is critical to perform the adequate weighting within the positioning algorithm. This is then further evaluated with a stand-alone localization method based on mmWave observables in line-of-sight (LoS) conditions. First the error bounds of mmWave observables are analyzed via the derivation of Fisher Information Matrix (FIM). Then the mmWave localization method is evaluated via simulations, whose results show that the centimeter-level accuracies for UE localization are feasible. The advantage of multi-connectivity is clear and significant over the single base-station approach, nonetheless, the cost for coordination and coverage is higher.

## Figures and Tables

**Table 1:** GNSS elevation mask models for straight street scenarios.

Model	Elevation Mask Definition	Description	Application Scenario
ETSI [13]	Constant for a range of azimuths	Simple and partially accurate	Predefined urban canyon
Sand et al. [20]	Dependent on street width, building height, receiver location and azimuth	Flexible and accurate	Receiver at any location of any symmetrical street
Proposed [21]	Dependent on street width, building height and azimuth	Generic and accurate	Receiver at the center of any symmetrical street

**Table 2:** Hybrid scenario definition based on cellular network density and GNSS elevation mask.

Scenario	Average Distance to 3 Closest BSs (m)	Channel Model	Max. Elevation Mask (°)
Rural	$d_{\mathrm{av},3\mathrm{BSs}}>500$	RMa	5
Suburban	$200<d_{\mathrm{av},3\mathrm{BSs}}\leq 500$	UMa	15
Urban	$100<d_{\mathrm{av},3\mathrm{BSs}}\leq 200$	UMi	30
Urban	$50<d_{\mathrm{av},3\mathrm{BSs}}\leq 100$	UMi	50
Urban	$d_{\mathrm{av},3\mathrm{BSs}}\leq 50$	UMi	70

**Table 3:** 2D position error (in meters) and GDOP on the 95% of the cases with stand-alone cmWave ToA positioning as function of the number of BSs.

Scenario	$M_{\mathrm{BS}}=3$		$M_{\mathrm{BS}}=4$		$M_{\mathrm{BS}}=5$	
	$\varepsilon_{2D}$	${\mathrm{GDOP}}_{2D}$	$\varepsilon_{2D}$	${\mathrm{GDOP}}_{2D}$	$\varepsilon_{2D}$	${\mathrm{GDOP}}_{2D}$
Urban	100.8	>10	60.5	>10	20.4	4.4
Suburban	100.8	>10	100.4	>10	59.3	4.9
Rural	100.5	>10	59.6	>10	27.0	9.9

**Table 4:** 3D position error (in meters) and GDOP on the 95% of the cases for the GNSS positioning with differential or precise corrections and the hybrid GNSS and cmWave ToA positioning with and without LoS knowledge.

Scenario	GNSS (with Corrections)		Hybrid (LoS/NLoS)		Hybrid (Known LoS)	
	$\varepsilon_{3D}$	${\mathrm{GDOP}}_{3D}$	$\varepsilon_{3D}$	${\mathrm{GDOP}}_{3D}$	$\varepsilon_{3D}$	${\mathrm{GDOP}}_{3D}$
Urban, 5 BSs	8.5	7.2	10.8	1.6	6.0	4.7
Suburban, 4 BSs	4.9	2.5	26.9	1.5	4.7	2.4
Rural, 4 BSs	4.8	1.9	5.5	1.4	3.3	1.5

## Appendix A. CRLB on mmWave Channel Parameters

In this section, the derivation of the Fisher Information matrix related to the estimation of the channel parameters $\eta =[\tau_{0},\theta_{0},\phi_{0},h_{0}]$ for the LoS channel is provided. To begin with, let the matrix J denote the FIM given by

(A1) $$J=\;\frac{1}{\sigma^{2}}E_{a,\omega }\left\{ℜ\left\{\nabla^{H}y_{n,m}\nabla y_{n,m}\right\}\right\},$$

where $y_{n,m}$ is the received signal at the *m*-th receiving chain at the *n*-th subcarrier and $E_{a,\omega }$ indicates the expectation of a random variable with respect to the distribution of *a* (pilot symbol) and ω (frequency).

To compute the gradient $\nabla y_{n,m}$, the following partial derivatives are used

(A2) $$\begin{array}{cc} \frac{\partial }{\partial \tau_{0}}y_{n,m} & =-j\frac{\sqrt{P_{c}}}{\sigma }2\pi \Delta_{f}h_{0}ne^{-j2\pi n\Delta_{f}\tau_{0}}w_{m}^{H}H_{0}Fa_{n}, \end{array}$$

(A3) $$\begin{array}{cc} \frac{\partial }{\partial \theta_{0}}y_{n,m} & =\frac{\sqrt{P_{c}}}{\sigma }h_{0}e^{-j2\pi n\Delta_{f}\tau_{0}}w_{m}^{H}D_{r}\left(\theta_{0}\right)a_{R}\left(\theta_{0}\right)a_{T}^{H}\left(\phi_{0}\right)Fa_{n} \end{array}$$

(A4) $$\begin{array}{cc} \frac{\partial }{\partial \phi_{0}}y_{n,m} & =\frac{\sqrt{P_{c}}}{\sigma }h_{0}e^{-j2\pi n\Delta_{f}\tau_{0}}w_{m}^{H}a_{R}\left(\theta_{0}\right)a_{T}^{H}\left(\phi_{0}\right)D_{t}^{H}\left(\phi_{0}\right)Fa_{n}, \end{array}$$

(A5) $$\begin{array}{cc} \frac{\partial }{\partial h_{I0}}y_{n,m} & =\frac{\sqrt{P_{c}}}{\sigma }e^{-j2\pi n\Delta_{f}\tau_{0}}w_{m}^{H}H_{0}Fa_{n}, \end{array}$$

(A6) $$\begin{array}{cc} \frac{\partial }{\partial h_{Q0}}y_{n,m} & =j\frac{\sqrt{P_{c}}}{\sigma }e^{-j2\pi n\Delta_{f}\tau_{0}}w_{m}^{H}H_{0}Fa_{n}, \end{array}$$

where $P_{c}$ and $\sigma^{2}$ is transmit power and noise per subcarrier and $h_{0I}$ and $h_{0Q}$ are the in-phase and quadrature term of the complex number *h*.

Then each component of the FIM is given by (in following we use $a_{r}$ and $a_{t}$ to shorten the notation of $a_{r}\left(\theta \right),a_{t}\left(\phi \right)$)

(A7) $$\begin{array}{c} J_{\tau ,\tau }^{n}=\frac{P_{c}}{\sigma^{2}}ℜ\left\{E_{a}\left\{\frac{\partial y_{n,m}^{H}}{\partial \tau_{0}}\frac{\partial y_{n,m}}{\partial \tau_{0}}\right\}\right\}=\frac{P_{c}}{\sigma^{2}}|h_{0}{|}^{2}{\left(2\pi \Delta_{f}\right)}^{2}n^{2}{|q_{w}∥}^{2}\mathrm{tr}\left(\Gamma_{f}\Sigma_{n}\right), \end{array}$$

with $\Gamma_{f}=F^{H}a_{T}a_{T}^{H}F$ and $\Sigma_{n}$ is the expected covariance of the transmitted symbols over the *n*-th sub-carrier, for instance, $\Sigma_{n}=I$ for QAM independent symbols, and note that the expectation in ω can be removed as all partial derivatives are independent of the frequency;

(A8) $$J_{\theta ,\theta }^{n}=\frac{P_{c}}{\sigma^{2}}ℜ\left\{E_{a}\left\{\frac{\partial y_{n,m}^{H}}{\partial \theta_{0}}\frac{\partial y_{n,m}}{\partial \theta_{0}}\right\}\right\}=\frac{P_{c}}{\sigma^{2}}|h_{0}{|}^{2}{∥{\dot{q}}_{w}∥}^{2}\mathrm{tr}\left(\Gamma_{f}\Sigma_{n}\right),$$

with ${\dot{q}}_{w}=w^{H}D_{r}a_{r}$ and $\Gamma_{f}=F^{H}a_{T}a_{T}^{H}F$;

(A9) $$J_{\phi ,\phi }^{n}=\frac{P_{c}}{\sigma^{2}}ℜ\left\{E_{a}\left\{\frac{\partial y_{n,m}^{H}}{\partial \phi_{0}}\frac{\partial y_{n,m}}{\partial \phi_{0}}\right\}\right\}=\frac{P_{c}}{\sigma^{2}}|h_{0}{|}^{2}{∥q_{w}∥}^{2}\mathrm{tr}\left(\Gamma_{df}\Sigma_{n}\right),$$

with $\Gamma_{df}=F^{H}D_{t}a_{T}a_{T}^{H}D_{t}^{H}F$;

(A10) $$J_{h_{I0},h_{I0}}^{n}=\frac{P_{c}}{\sigma^{2}}ℜ\left\{E_{a}\left\{\frac{\partial y_{n,m}^{H}}{\partial h_{I0}}\frac{\partial y_{n,m}}{\partial h_{I0}}\right\}\right\}=\frac{P_{c}}{\sigma^{2}}{|q_{w}∥}^{2}\mathrm{tr}\left(\Gamma_{f}\Sigma_{n}\right),$$

(A11) $$J_{h_{Q0},h_{Q0}}^{n}=J_{h_{I0},h_{I0}}^{n},$$

(A12) $$J_{\theta ,\phi }^{n}=\frac{P_{c}}{\sigma^{2}}ℜ\left\{E_{a}\left\{\frac{\partial y_{n,m}^{H}}{\partial \theta_{0}}\frac{\partial y_{n,m}}{\partial \phi_{0}}\right\}\right\}=\frac{P_{c}}{\sigma^{2}}{\left|h_{0}\right|}^{2}ℜ\left\{q_{w}^{*}{\dot{q}}_{w}\mathrm{tr}\left(\Gamma_{rt}\Sigma_{n}\right)\right\},$$

with

$$\Gamma_{rt}=F^{H}D_{t}a_{T}a_{T}^{H}F;$$

(A13) $$\begin{array}{cc} J_{\theta ,h_{I}}^{n} & =\frac{P_{c}}{\sigma^{2}}ℜ\left\{E_{a}\left\{\frac{\partial y_{n,m}^{H}}{\partial \theta_{0}}\frac{\partial y_{n,m}}{\partial h_{I}}\right\}\right\}\\ & =\frac{P_{c}}{\sigma^{2}}ℜ\left\{E_{a}\left\{c_{0}^{*}a_{n}^{H}F^{H}a_{T}\left(\phi_{0}\right)w_{m}^{H}D_{r}\left(\theta_{0}\right)a_{R}\left(\theta_{0}\right)w_{m}^{H}H_{0}Fa_{n}\right\}\right\}\\ & =\frac{P_{c}}{\sigma^{2}}ℜ\left\{c^{*}{\dot{q}}_{w}^{*}q_{w}\mathrm{tr}\left(\Gamma_{f}\Sigma_{n}\right)\right\}, \end{array}$$

(A14) $$\begin{array}{cc} J_{\theta ,h_{Q}}^{n} & =\frac{P_{c}}{\sigma^{2}}ℜ\left\{E_{a}\left\{\frac{\partial y_{n,m}^{H}}{\partial \theta_{0}}\frac{\partial y_{n,m}}{\partial h_{Q}}\right\}\right\}\\ & =\frac{P_{c}}{\sigma^{2}}ℜ\left\{E_{a}\left\{jc_{0}^{*}a_{n}^{H}F^{H}a_{T}\left(\phi_{0}\right)w_{m}^{H}D_{r}\left(\theta_{0}\right)a_{R}\left(\theta_{0}\right)w_{m}^{H}H_{0}Fa_{n}\right\}\right\}\\ & =\frac{P_{c}}{\sigma^{2}}ℜ\left\{jh_{0}^{*}{\dot{q}}_{w}^{*}q_{w}\mathrm{tr}\left(\Gamma_{f}\Sigma_{n}\right)\right\}, \end{array}$$

(A15) $$\begin{array}{cc} J_{\phi ,h_{I}}^{n} & =\;\frac{P_{c}}{\sigma^{2}}ℜ\left\{E_{a}\left\{\frac{\partial y_{n,m}^{H}}{\partial \phi_{0}}\frac{\partial y_{n,m}}{\partial h_{I}}\right\}\right\}\\ & =\frac{P_{c}}{\sigma^{2}}ℜ\left\{E_{a}\left\{c_{0}^{*}a_{n}^{H}F^{H}D_{t}\left(\phi_{0}\right)a_{T}\left(\phi_{0}\right)a_{R}^{H}\left(\theta_{0}\right)w_{m}w_{m}^{H}H_{0}Fa_{n}\right\}\right\}\\ & =\frac{P_{c}}{\sigma^{2}}{\left|q_{w}\right|}^{2}ℜ\left\{\mathrm{tr}\left(\Gamma_{tI}\Sigma_{n}\right)\right\}, \end{array}$$

(A16) $$\begin{array}{cc} J_{\phi ,h_{Q}}^{n} & =\;\frac{P_{c}}{\sigma^{2}}ℜ\left\{E_{a}\left\{\frac{\partial y_{n,m}^{H}}{\partial \phi_{0}}\frac{\partial y_{n,m}}{\partial h_{Q}}\right\}\right\}\\ & =\frac{P_{c}}{\sigma^{2}}ℜ\left\{E_{a}\left\{jc_{0}^{*}a_{n}^{H}F^{H}D_{t}\left(\phi_{0}\right)a_{T}\left(\phi_{0}\right)a_{R}^{H}\left(\theta_{0}\right)w_{m}w_{m}^{H}H_{0}Fa_{n}\right\}\right\}\\ & =\frac{P_{c}}{\sigma^{2}}{\left|q_{w}\right|}^{2}ℜ\left\{\mathrm{tr}\left(\Gamma_{tQ}\Sigma_{n}\right)\right\}. \end{array}$$

Summarizing all the above derivations, the non-zero FIM components are given by

(A17) $$J_{\tau ,\tau }^{n}=\frac{P_{c}}{\sigma^{2}}|h_{0}{|}^{2}{\left(2\pi \Delta_{f}\right)}^{2}n^{2}{∥q_{w}∥}^{2}\mathrm{tr}\left(\Gamma_{f}\Sigma_{n}\right)$$

(A18) $$J_{\phi^{r},\phi^{r}}^{n}=\frac{P_{c}}{\sigma^{2}}|h_{0}{|}^{2}{∥{\dot{q}}_{w}∥}^{2}\mathrm{tr}\left(\Gamma_{f}\Sigma_{n}\right)$$

(A19) $$J_{\phi^{t},\phi^{t}}^{n}=\frac{P_{c}}{\sigma^{2}}|h_{0}{|}^{2}{∥q_{w}∥}^{2}\mathrm{tr}\left(\Gamma_{df}\Sigma_{n}\right)$$

(A20) $$J_{h_{I},h_{I}}^{n}=\frac{P_{c}}{\sigma^{2}}{∥q_{w}∥}^{2}\mathrm{tr}\left(\Gamma_{f}\Sigma_{n}\right)$$

(A21) $$J_{h_{R},h_{R}}^{n}=\frac{P_{c}}{\sigma^{2}}{∥q_{w}∥}^{2}\mathrm{tr}\left(\Gamma_{f}\Sigma_{n}\right)$$

(A22) $$J_{\phi^{r},\phi^{t}}^{n}=\frac{P_{c}}{\sigma^{2}}{\left|h_{0}\right|}^{2}ℜ\left\{q_{w}^{*}{\dot{q}}_{w}\mathrm{tr}\left(\Gamma_{rt}\Sigma_{n}\right)\right\}$$

(A23) $$J_{\phi^{r},h_{I}}^{n}=\frac{P_{c}}{\sigma^{2}}ℜ\left\{h_{0}^{*}{\dot{q}}_{w}^{*}q_{w}\mathrm{tr}\left(\Gamma_{f}\Sigma_{n}\right)\right\}$$

(A24) $$J_{\phi^{r},h_{R}}^{n}=\frac{P_{c}}{\sigma^{2}}ℜ\left\{jh_{0}^{*}{\dot{q}}_{w}^{*}q_{w}\mathrm{tr}\left(\Gamma_{f}\Sigma_{n}\right)\right\}$$

(A25) $$J_{\phi^{t},h_{I}}^{n}=\frac{P_{c}}{\sigma^{2}}{∥q_{w}∥}^{2}ℜ\left\{h_{0}^{*}\mathrm{tr}\left(\Gamma_{rt}\Sigma_{n}\right)\right\}$$

(A26) $$J_{\phi^{t},h_{R}}^{n}=\frac{P_{c}}{\sigma^{2}}{∥q_{w}∥}^{2}ℜ\left\{jh_{0}^{*}\mathrm{tr}\left(\Gamma_{rt}\Sigma_{n}\right)\right\}$$

with

(A27) $$a_{R}\left(\phi^{r}\right)={\left[1,e^{j\pi \sin(\theta )},\cdots ,e^{j\pi \left(M-1\right)\sin\left(\phi^{r}\right)}\right]}^{T}$$

(A28) $$a_{T}\left(\phi^{t}\right)={\left[1,e^{j\pi \sin(\phi )},\cdots ,e^{j\pi \left(N-1\right)\sin\left(\phi^{t}\right)}\right]}^{T}$$

(A29) $$D_{R}\left(\phi^{r}\right)=j\pi \cos\left(\theta \right)\mathrm{diag}\left({\left[0,\cdots ,\left(M-1\right)\right]}^{T}\right)a_{R}\left(\phi^{r}\right)$$

(A30) $$D_{T}\phi^{t})=j\pi \cos\left(\phi \right)\mathrm{diag}\left({\left[0,\cdots ,\left(N-1\right)\right]}^{T}\right)a_{T}\left(\phi^{t}\right)$$

(A31) $$q_{w}=w^{H}a_{R}\left(\phi^{r}\right)$$

(A32) $${\dot{q}}_{w}=w^{H}D_{r}\left(\phi^{r}\right)a_{R}\left(\phi^{r}\right)$$

(A33) $$\Sigma_{n}=E_{a}\left\{a_{n}a_{n}^{H}\right\}$$

(A34) $$\Gamma_{f}=F^{H}a_{T}\left(\phi^{t}\right)a_{T}^{H}\left(\phi^{t}\right)F$$

(A35) $$\Gamma_{df}=F^{H}D_{t}\left(\phi^{t}\right)a_{T}\left(\phi^{t}\right)a_{T}^{H}\left(\phi^{t}\right)D_{t}^{H}F$$

(A36) $$\Gamma_{rt}=F^{H}D_{t}\left(\phi^{t}\right)a_{T}\left(\phi^{t}\right)a_{T}^{H}\left(\phi^{t}\right)F$$
